# Decadal trends and regional disparities in tuberculosis burden: a comprehensive analysis of global, African, and Southeast Asian data from the GBD 1990–2021

**DOI:** 10.3389/fpubh.2025.1467509

**Published:** 2025-08-04

**Authors:** Shiwei Xie, Heng Xiao, Lei Xu, Fan Zhang, Mingwei Luo

**Affiliations:** ^1^Panzhihua Central Hospital, Panzhihua, Sichuan, China; ^2^Department of Orthopedic Surgery, The First Affiliated Hospital of Kunming Medical University, Kunming, Yunnan, China

**Keywords:** tuberculosis, incidence, mortality, disability-adjusted life years, global burden of disease

## Abstract

**Background:**

Tuberculosis (TB), an infectious disease caused by *Mycobacterium tuberculosis*, remains a major global public health challenge, particularly in developing countries. Despite a global reduction in TB incidence from 2015 to 2020, the disease continues to be prevalent, with 9.4 million new cases and 1.35 million deaths reported in 2021. This study aims to assess the global, regional, and national burden of TB, with a specific focus on Africa and Southeast Asia, using data from the Global Burden of Disease Study.

**Methods:**

Data from the Global Burden of Disease 2021 (GBD 2021) study were used to evaluate TB incidence, prevalence, mortality, and disability-adjusted life years (DALYs) from 1990 to 2021. Statistical analyses were conducted using R software and Joinpoint Regression Program to identify trends in age-standardized incidence rate (ASIR), age-standardized mortality rate (ASMR), and age-standardized DALY rate (ASDR). The annual percentage change (APC) was calculated to assess the significance of temporal trends.

**Results:**

From 1990 to 2021, global age-standardized rates of TB declined markedly, with ASIR decreasing from 173.0 to 103.0 per 100,000, ASMR from 40.0 to 14.0, and ASDR from 1,650.6 to 580.3. Although incident case numbers slightly declined globally, absolute numbers increased in Africa and Southeast Asia, despite reductions in standardized rates. The disease burden has shifted from younger to older age groups, reflecting population aging. Males consistently exhibited a higher burden than females, though sex disparities narrowed over time. Joinpoint regression confirmed sustained declines in all indicators, particularly in Africa and Southeast Asia. Projections to 2040 suggest continued reductions and convergence in burden across regions. Spatial analyses identified persistent high-burden clusters in sub-Saharan Africa and Southeast Asia, despite overall global improvement.

**Conclusion:**

TB remains a significant public health issue, especially in Africa and Southeast Asia. While global incidence and mortality have decreased, persistent regional disparities call for more targeted interventions. Ongoing global efforts are essential to further reduce TB-related morbidity and mortality.

## Background

Tuberculosis (TB), an infectious disease caused by *Mycobacterium tuberculosis*, remains a major contributor to the global burden of disease, particularly in developing countries. Although it primarily affects the lungs, extrapulmonary manifestations—such as skeletal TB, gastrointestinal TB and tuberculous meningitis—are also observed. According to the GBD 2021 modeling framework, an estimated 9.40 million (95% UI 8.36–10.5) new TB cases and 1.35 million (1.23–1.52) TB deaths occurred in 2021 (1), underscoring that TB continues to pose a significant public-health challenge. The burden of TB is influenced by a complex interplay of factors, including socioeconomic status, healthcare access, genetic predisposition and environmental conditions (2). Consequently, efforts to control TB encompass strategies from early diagnosis and treatment to public-health interventions aimed at reducing transmission and addressing social determinants of health.

To further reduce TB morbidity and mortality, countries heavily impacted by TB must systematically implement evidence-based prevention and treatment strategies and evaluate their cost-effectiveness. Moreover, advancements in diagnostic and surgical techniques have altered the epidemiological patterns of TB (3). Together, these developments make the ongoing monitoring of TB trends essential for effective public-health planning. Current GBD assessments have predominantly provided global and broad regional estimates of TB incidence, mortality and DALYs from 1990 onward (1, 4, 5), explored associations with socioeconomic indicators (6–8), and projected future trajectories for selected regions (9, 10). However, many of these analyses overlook the nuanced, country- and subregion-specific circumstances that drive local TB dynamics. Public-health discussions have particularly emphasized sub-Saharan Africa and South-East Asia—regions that together accounted for nearly half of incident TB cases and more than half of TB deaths in 2021 (11, 12). Accordingly, we present a comparative analysis at the global level, and for the WHO African Region and the South-East Asia Region as defined by the GBD study—enabling us to disentangle universal from region-specific drivers and to target recommendations where progress toward End TB targets is most needed. While some studies (13) have examined TB trends in these regions, focusing on metrics such as mortality and DALYs, a thorough and detailed investigation into TB progression among these populations remains limited. Using the most recent GBD estimates for 1990–2021, we performed a comprehensive analysis of tuberculosis incidence, prevalence, mortality and DALYs at global scale and for two high-burden regions—sub-Saharan Africa and South-East Asia—down to the national, age- and sex-specific level. We applied joinpoint regression and related trend-analysis methods were applied to quantify annual changes and elucidate long-term trajectories, overcoming the limitations of earlier studies that examined single indicators or short time frames. By pinpointing where and in whom progress toward the WHO End TB goal of a 90% mortality reduction by 2030 is lagging, our findings provide an evidence base for policymakers to refine resource allocation, implement age-tailored screening and deploy region-specific intervention packages. The institutional ethics committee granted an exemption for this study, as it did not require approval, given that the data from the 2021 GBD is publicly available.

## Methods

### Data source

We drew all TB data from the Global Burden of Disease Study 2021, a worldwide collaborative initiative led by the Institute for Health Metrics and Evaluation that integrates contributions from over 12,000 researchers across more than 160 countries to quantify health trends over time. Using the publicly accessible GBD Results Portal^1^, we queried: Cause = “Tuberculosis”; specifically, we extracted the parent-level cause “tuberculosis,” which provides aggregated estimates for HIV-negative drug-susceptible TB, multidrug-resistant TB (MDR-TB), extensively drug-resistant TB (XDR-TB), and also includes latent tuberculosis infection (LTBI); Measure = “Deaths, DALYs, YLDs, YLLs, Incidence”; Metric = “Number, Rate, Percent”; Location = “Global, Africa, South-East Asia”; Age = “All”; Sex = “Both”; and Year = 1990–2021. For this study we used the GBD definition of ‘Southeast Asia,’ which comprises Cambodia, Indonesia, Lao People’s Democratic Republic, Malaysia, Maldives, Mauritius, Myanmar, Philippines, Seychelles, Sri Lanka, Thailand, Timor-Leste and Viet Nam. GBD generates age-, sex-, year- and location-specific estimates with two core modeling platforms. DisMod-MR 2.1 (a Bayesian meta-regression) reconciles routine case notifications, population-based prevalence surveys, tuberculin-survey data and cause-specific mortality to produce internally consistent incidence and point prevalence estimates, even where primary data are sparse. Cause-specific mortality is estimated with the Cause of Death Ensemble model (CODEm), which aggregates thousands of mixed-effects models built from vital-registration, verbal-autopsy, sample-registration and mortality-surveillance inputs after redistribution of non-informative ICD codes. DALYs are computed as the sum of years of life lost (YLL = number of deaths × standard life expectancy at age of death) and years lived with disability (YLD = prevalence × disability weight; DW for active pulmonary TB = 0.331), with YLDs adjusted for multimorbidity by multiplicative simulation. All rates are directly age-standardized to the GBD reference population, and 95% uncertainty intervals (UIs) are derived from 1,000 posterior draws, propagating uncertainty from every modeling step. The GBD 2021 framework models TB as a single HIV-negative cause; HIV-associated TB is therefore outside the scope of the present analysis.

### Statistical analysis

The analysis was conducted using R (version 4.4.0) for data management and visualization and Joinpoint (version 5.1.0; National Cancer Institute, Rockville, MD, United States) for trend estimation. A two-sided *p* < 0.05 was considered statistically significant. We evaluated both crude and age-standardized measures of tuberculosis burden—incidence, prevalence, mortality and DALY—reporting crude incidence rate (CIR), crude mortality rate (CMR) and crude DALY rate (CDR) alongside ASIR, ASMR and ASDR for each age group. Joinpoint regression was used to estimate the average annual percentage change (AAPC) and its 95% confidence interval (CI) for each measure. For age-standardized rates, we fitted a log-linear model of the form ln(*y*) = *α + βx+ε*, where y is the age-standardized rate, *x* is calendar year, and *α,β* and *ε* are regression parameters and error term, respectively. The APC was calculated as APC = 100 × (exp(*β*) − 1), with its 95% CI derived from the variance of *β*. Additionally, the 95% confidence interval (CI) for the APC estimate can be computed. If the 95% CI of the APC estimate is greater than 0, it indicates an increasing trend in the age-standardized indicator. Conversely, if it is less than 0, it suggests a decreasing trend. A CI that includes 0 suggests a stable trend in the indicator over time. For each ARIMA model used in the trend analyses of TB incidence and mortality (by sex and region), we report standard model diagnostics including: Akaike Information Criterion (AIC), corrected AIC (AICc), Bayesian Information Criterion (BIC), standard errors of model coefficients, residual variance (σ^2^), and first-order autocorrelation (ACF1) of residuals. Additionally, model accuracy was assessed using training set performance metrics such as mean error (ME), root mean square error (RMSE), mean absolute error (MAE), mean absolute percentage error (MAPE), and mean absolute scaled error (MASE). A summary of these diagnostics is provided in Supplementary Table S1 to ensure transparency and methodological rigor.

## Results

### Global and regional trends in tuberculosis burden from 1990 to 2021

From 1990 to 2021, all-age incident TB cases decreased globally from 8.60 million (95% CI: 7.53–9.85 million) to 8.41 million (7.52–9.39 million), while the ASIR declined from 173.0 to 103.0 per 100,000 (AAPC = −1.65%). In Africa, incident cases rose from 1.86 million to 2.28 million, but ASIR dropped from 384.9 to 206.5 (AAPC = −1.98%), indicating a substantial population-adjusted improvement. South-East Asia showed a modest increase in absolute cases but a significant ASIR reduction from 314.1 to 181.4 (AAPC = −1.88%) (Table 1).

**Table 1:** All-age cases and age-standardized incidence, mortality, and DALYs rates and corresponding AAPC of TB in Global, Africa and Southeast Asia in 1990 and 2021.

Location	Measure	1990		2021		1990–2021 AAPC
		All-ages cases	Age-standardized rates per 100,000 people	All-ages cases	Age-standardized rates per 100,000 people	
		*n* (95% CI)	*n* (95% CI)	*n* (95% CI)	*n* (95% CI)	*n* (95% CI)
Global	Incidence	8,598,520 (7528228–9,854,794)	173.032 (152.882–198.712)	8,407,133 (7519793–9,393,767)	102.996 (92.21–114.906)	−1.6549 (−1.7397 – −1.5701)
	Deaths	1,778,869 (1532822–1,980,801)	39.991 (34.159–44.763)	1,162,796 (1050008–1,313,985)	13.957 (12.61–15.717)	−3.0922 (−3.1962 – −2.9881)
	DALYs	82,679,773 (73004989–91,407,746)	1650.591 (1457.637–1824.716)	46,977,463 (42482994–52,463,556)	580.263 (522.374–649.816)	−3.3163 (−3.4259 – −3.2065)
Africa	Incidence	1,858,276 (1670236–2,059,099)	384.934 (349.819–425.023)	2,278,940 (2020461–2,548,091)	206.533 (184.223–227.85)	−1.9753 (−2.0183 – −1.9323)
	Deaths	448,336 (378469–537,012)	118.345 (97.929–142.243)	380,166 (319771–450,268)	49.934 (42.69–58.362)	−2.4486 (−2.539 – −2.3582)
	DALYs	23,486,586 (19979095–27,482,420)	4348.229 (3700.635–5140.137)	17,389,960 (14226267–20,673,165)	1670.091 (1412.337–1957.915)	−3.0466 (−3.1199 – −2.9732)
Southeast Asia	Incidence	1,166,408 (1050844–1,289,088)	314.14 (285.196–345.809)	1,264,295 (1148247–1,395,423)	181.415 (164.654–198.796)	−1.7454 (−1.7865 – −1.7042)
	Deaths	252,323 (201815–288,293)	87.278 (68.3–100.449)	170,555 (147940–199,977)	27.254 (23.533–31.563)	−3.4673 (−3.5321 – −3.4025)
	DALYs	11,355,171 (9452930–12,691,803)	3012.77 (2471.446–3395.487)	6,169,600 (5438974–7,066,376)	895.707 (793.04–1020.931)	−3.8107 (−3.9214 – −3.6998)

Global TB deaths decreased from 1.78 million (1.53–1.98 million) to 1.16 million (1.05–1.31 million), with ASMR falling from 40.0 to 14.0 per 100,000 (AAPC = −3.09%). Africa and South-East Asia both showed significant declines, with ASMR dropping from 118.3 to 49.9 and from 87.3 to 27.3 per 100,000, respectively.

DALYs globally decreased from 82.7 million to 47.0 million, while the ASDR fell from 1650.6 to 580.3 (AAPC = −3.32%). The largest absolute reduction in DALYs occurred in Southeast Asia, whereas Africa experienced a smaller decline in DALY rates but still retained the highest per-capita burden in 2021. These patterns demonstrate that although TB burden has decreased overall, disparities persist across regions and metrics (Table 1).

### Global spatial distribution of tuberculosis burden in 1990 and 2021

**Figure 1:**
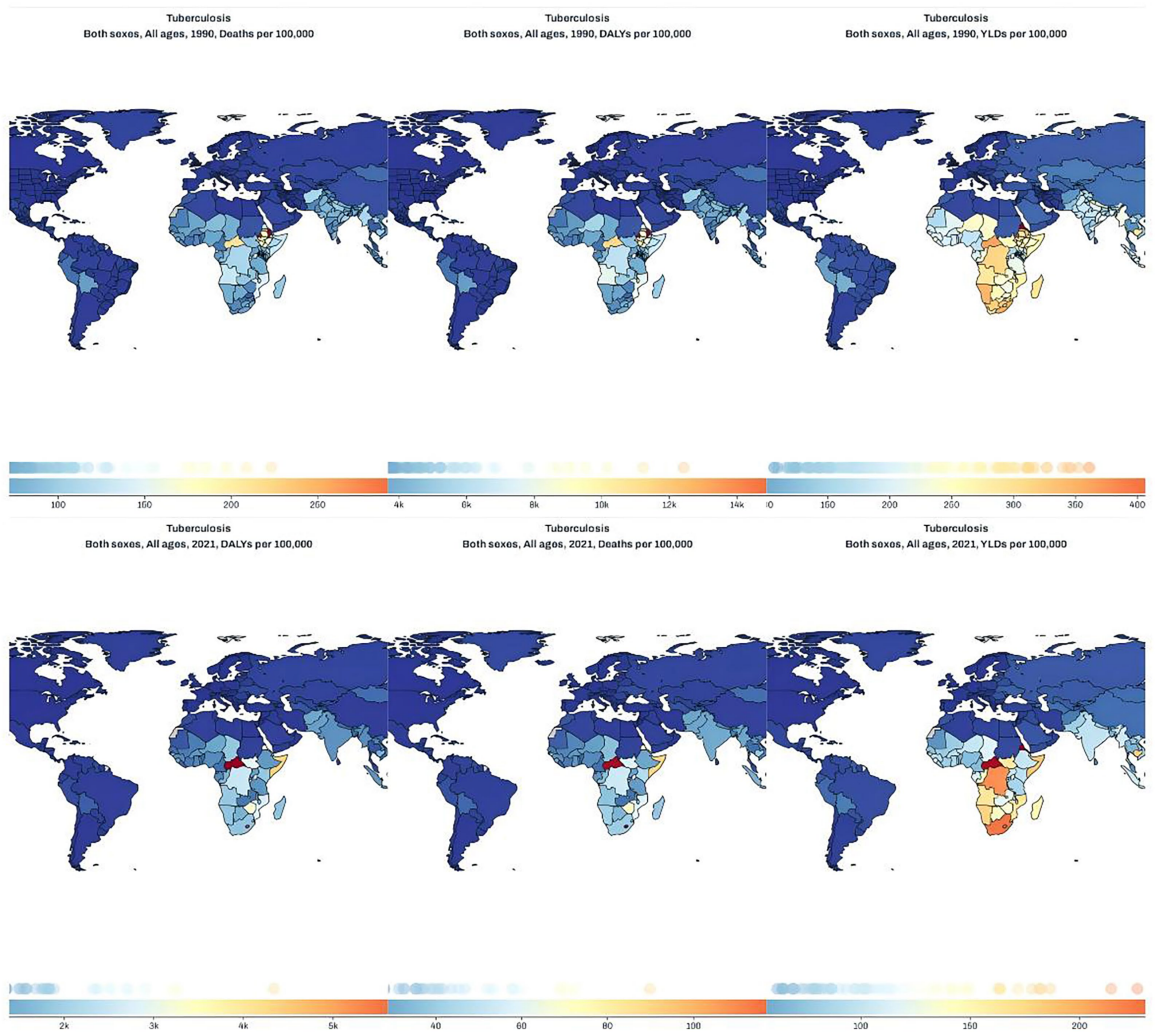
Global distribution maps of tuberculosis deaths (left), DALYs (middle), and YLDs (right) per 100,000 population in 1990 (top row) and 2021 (bottom row) across all ages and both sexes.

The global distribution of tuberculosis burden, as illustrated in Figure 1, reveals substantial geographic and temporal disparities between 1990 and 2021. In 1990, the highest rates of TB-related deaths and DALYs per 100,000 population were concentrated in sub-Saharan Africa, with particularly elevated levels observed in central and eastern regions. Several countries in these areas exceeded 150 deaths or 12,000 DALYs per 100,000, indicating severe health loss. YLDs were also elevated in parts of Africa and Southeast Asia, though to a lesser extent than mortality and overall DALYs. By 2021, TB mortality and DALY rates had declined markedly in most world regions, reflecting global progress in TB prevention and treatment. However, certain countries—such as the Democratic Republic of the Congo, Nigeria, and Mozambique—remained high-burden zones, with death rates above 60 and DALYs surpassing 4,000 per 100,000. Meanwhile, YLDs remained persistently high across eastern and southern Africa, suggesting ongoing challenges in reducing TB-related disability despite improvements in mortality. These spatial trends, as visualized in Figure 1, highlight both the successes and the persistent inequalities in the global fight against tuberculosis.

### Joinpoint regression analysis of the burden of TB in Africa, Southeast Asia and worldwide

**Figure 2:**
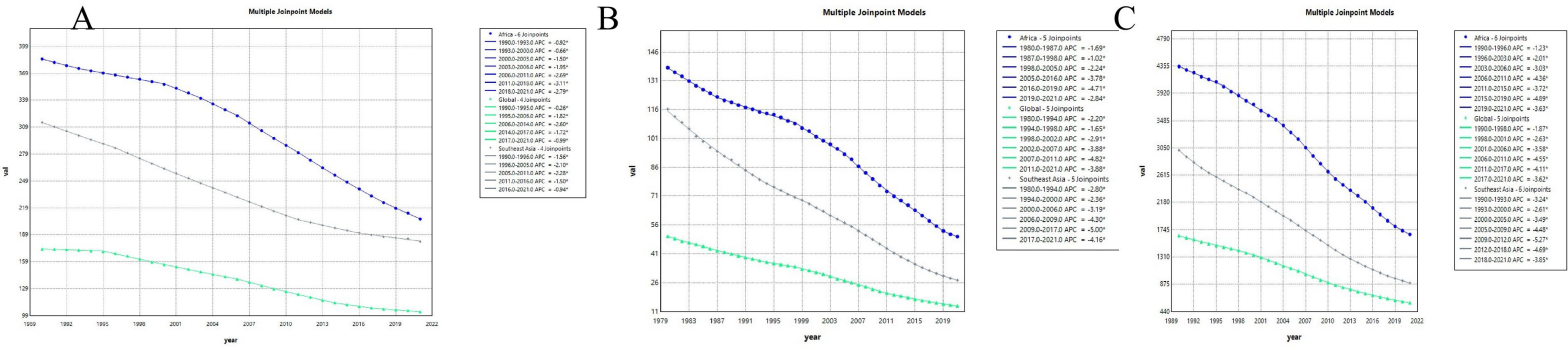
Joinpoint regression analysis of tuberculosis burden trends from 1990 to 2021 across Africa, Southeast Asia, and globally. **(A)** Incidence; **(B)** mortality; **(C)** DALYs.

The joinpoint regression analysis of age-standardized disease-burden metrics revealed consistent declines across Africa, the GBD South-East Asia Region, and the globe during 1990–2021 (Figures 2A–C). In Africa, the ASIR fell throughout the period, with the steepest annual percentage change (APC = −3.11%) observed from 2011 to 2018 (Figure 2A). The ASMR in Africa dropped markedly between 1980 and 2021, with the fastest decline during 2016–2019 (APC = −4.71%); globally, the largest reduction in ASMR occurred in 2007–2011 (APC = −4.82%). In South-East Asia, ASMR declined most sharply between 2009 and 2017 (APC = −5.00%; Figure 2B). Finally, age-standardized DALY rates decreased significantly in Africa from 2015 to 2019 (APC = −4.89%), globally from 2006 to 2010 (APC = −4.55%), and in South-East Asia from 2009 to 2012 (APC = −5.27%; Figure 2C).

### Trend comparison of ASIR, ASMR, and ASDR of TB in Africa, Southeast Asia and global

**Figure 3:**
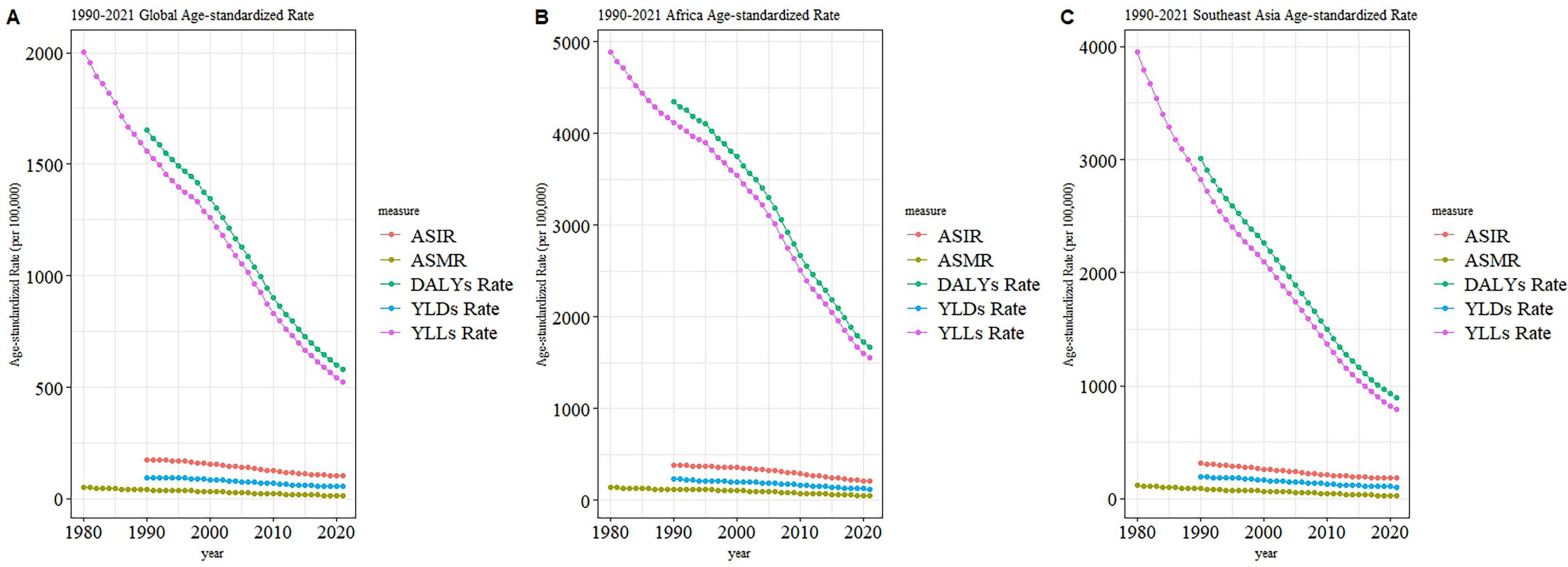
Age-standardized rates of tuberculosis indicators from 1990 to 2021 by region. **(A)** Global; **(B)** Africa; **(C)** Southeast Asia. Indicators include ASIR, ASMR, DALYs, YLDs, and YLLs.

Figure 3 illustrates trends in the ASIR, ASMR, DALYs rate, YLLs rate, and YLDs rate for tuberculosis globally (A), in Africa (B), and Southeast Asia (C) from 1990 to 2021. Overall, YLLs and DALYs rates in all three regions demonstrated significant declining trends, while the changes in YLDs, ASIR, and ASMR were relatively minor and stable. Among the three regions, Africa exhibited notably higher initial values and greater reductions in YLLs and DALYs rates compared to the global average and Southeast Asia. The trends in Southeast Asia closely resembled the global averages, although Southeast Asia began with higher initial values and showed larger reductions. Specifically, from 1990 to 2021, YLLs in Africa decreased from over 4,500 per 100,000 to approximately 1,500 per 100,000; in Southeast Asia, from nearly 4,000 per 100,000 to below 1,000 per 100,000; and globally, from around 2,000 per 100,000 to below 500 per 100,000. Both ASIR and ASMR exhibited minor fluctuations and no significant downward trends across all three regions, indicating that the decline in tuberculosis burden primarily resulted from substantial reductions in fatal disease burden (YLLs), rather than from significant decreases in new cases or mortality rates.

### Burden of TB in different age groups in Africa in 1990 and 2021

**Figure 4:**
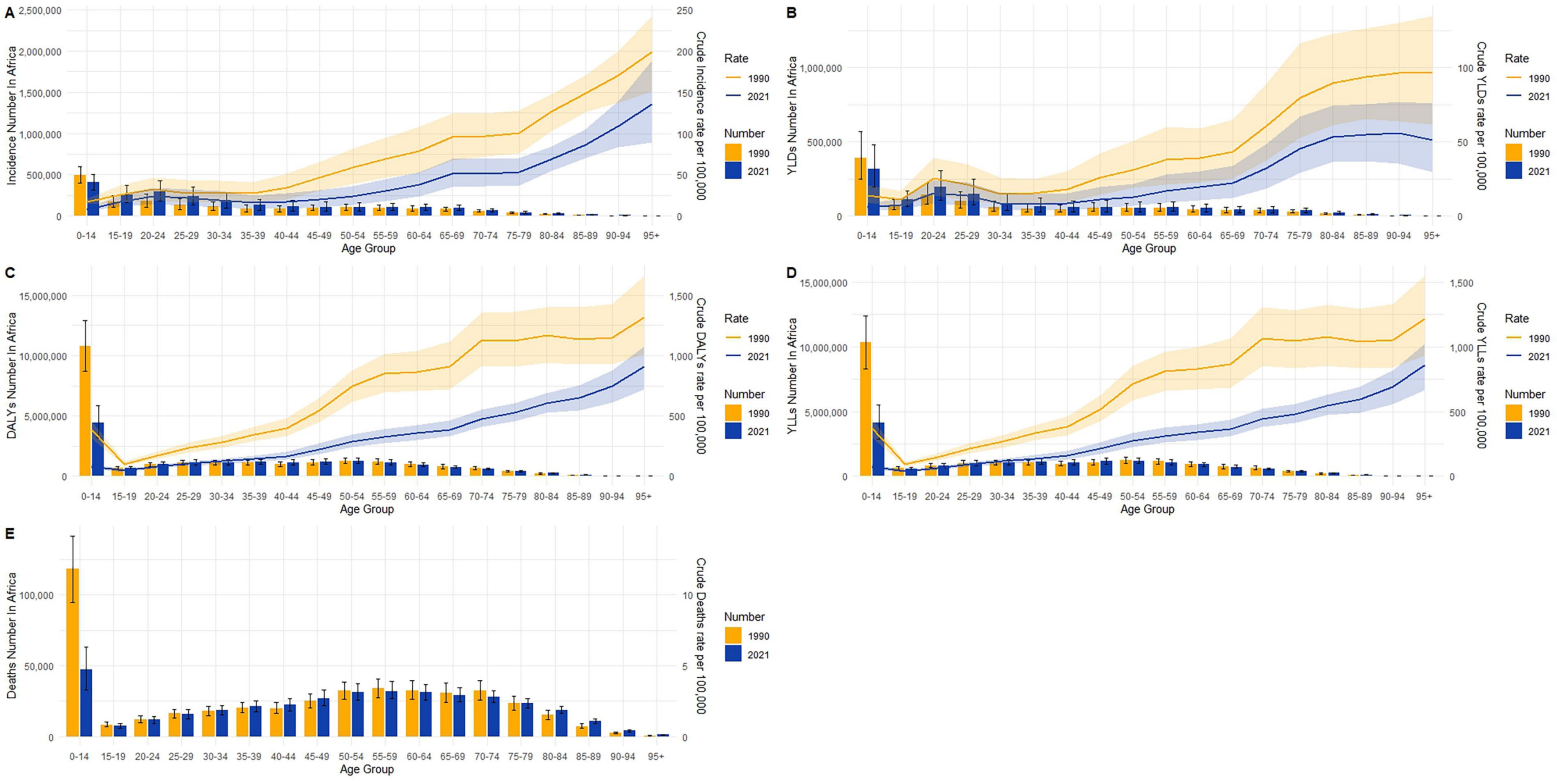
Age-specific crude rates and absolute numbers of tuberculosis burden in Africa in 1990 and 2021. **(A)** Incidence; **(B)** YLDs; **(C)** DALYs; **(D)** YLLs; **(E)** deaths.

Figure 4 displays age-specific distributions of crude rates and absolute numbers for multiple disease burden indicators in Africa in 1990 and 2021, including incidence (A), YLD (B), DALYs (C), YLLs, (D), and deaths (E). In both years, the 0–14 age group accounted for the highest burden across most indicators, particularly in 1990, when the numbers and rates of DALYs, YLLs, and deaths were markedly elevated. Although crude rates for all indicators generally decreased from 1990 to 2021 across all age groups, absolute numbers increased or remained stable in older age categories, reflecting population growth and demographic shifts. Notably, incidence in 2021 showed significant increases in absolute counts among individuals aged ≥60 years, despite lower rates compared to 1990. Similarly, YLDs and DALYs (B–C) exhibited a rightward shift in burden, with rising numbers and crude rates in older age groups, especially beyond age 60. The YLLs (D) and death counts (E) showed substantial reductions in the 0–14 age group, yet modest increases were observed in older populations. These patterns indicate a demographic transition in the distribution of disease burden in Africa, with declining early-age mortality but increasing chronic burden in older adults.

### Burden of TB in different age groups in Southeast Asia in 1990 and 2021

**Figure 5:**
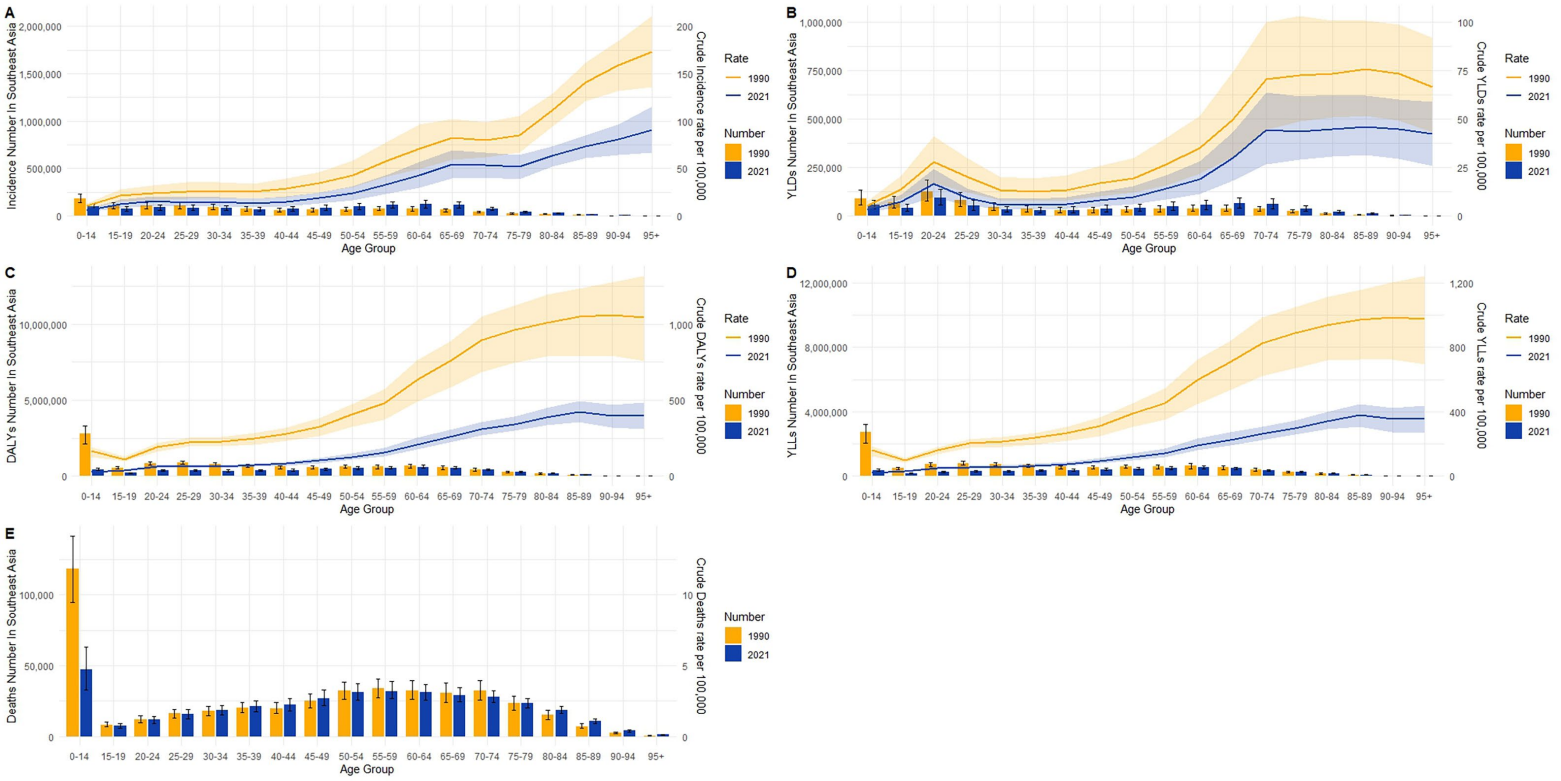
Age-specific crude rates and absolute numbers of tuberculosis burden in Southeast Asia in 1990 and 2021. **(A)** Incidence; **(B)** YLDs; **(C)** DALYs; **(D)** YLLs; **(E)** deaths.

Age-specific distributions of crude rates and absolute numbers in Southeast Asia revealed substantial shifts between 1990 and 2021 across multiple burden metrics, as shown in Figures 5A–E. Crude incidence rates (Figure 5A) declined across nearly all age groups, most notably among children and young adults, while absolute numbers increased among individuals aged ≥60 years, reflecting population aging. YLDs (Figure 5B) displayed a modest reduction in crude rates across most age groups, yet the absolute number of YLDs rose steadily with age in 2021, surpassing levels observed in 1990 for older populations. A similar pattern was observed for DALYs (Figure 5C), with a marked decline in crude rates among those under 15 years, but increased burden in older age groups in both absolute and relative terms. For YLLs (Figure 5D), sharp reductions were seen in both rate and number for the 0–14 age group, while the burden shifted toward older adults over time. Deaths (Figure 5E) followed a comparable trend, with notable improvements among children but persistent or slightly increased mortality in older age strata. Together, these findings point to a clear epidemiological transition, characterized by declining early-life mortality and rising chronic morbidity and disability in aging populations.

### Burden of TB in different age groups in global in 1990 and 2021

**Figure 6:**
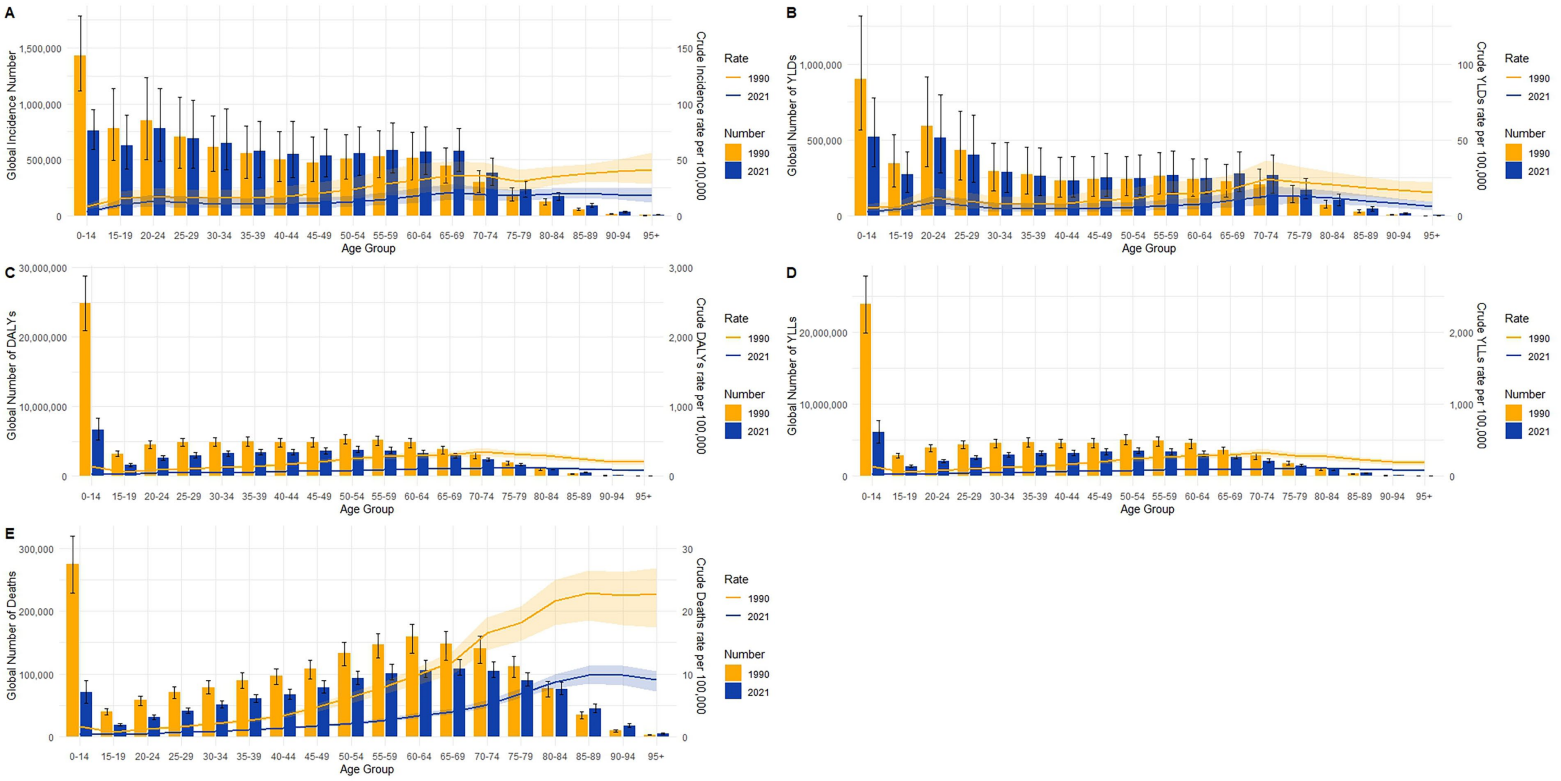
Age-specific crude rates and absolute numbers of tuberculosis burden globally in 1990 and 2021. **(A)** Incidence; **(B)** YLDs; **(C)** DALYs; **(D)** YLLs; **(E)** deaths.

Age-specific patterns in global burden estimates showed distinct shifts between 1990 and 2021 across all key indicators, as illustrated in Figures 6A–E. Crude incidence rates declined slightly across most age groups (Figure 6A), while the absolute numbers increased in older adults, particularly those aged 60 years and above. YLDs (Figure 6B) demonstrated modest reductions in crude rates for most ages, with numbers increasing slightly in older adult groups by 2021, indicating rising non-fatal morbidity. DALYs (Figure 6C) declined sharply in the 0–14 age group, primarily driven by a substantial drop in YLLs (Figure 6D), which showed a marked decrease in both rate and number among children. In contrast, the burden of YLLs and deaths gradually shifted toward older adults by 2021. Crude death rates (Figure 6E) decreased in early life but increased among the older adult, with the 85 + age group experiencing the highest mortality rates in 2021. These results reflect a global epidemiological transition characterized by reduced early-age mortality and increased disease burden due to aging populations and chronic conditions in later life.

### Gender disparities in the burden of TB in different age groups in 1990 and 2021

**Figure 7:**
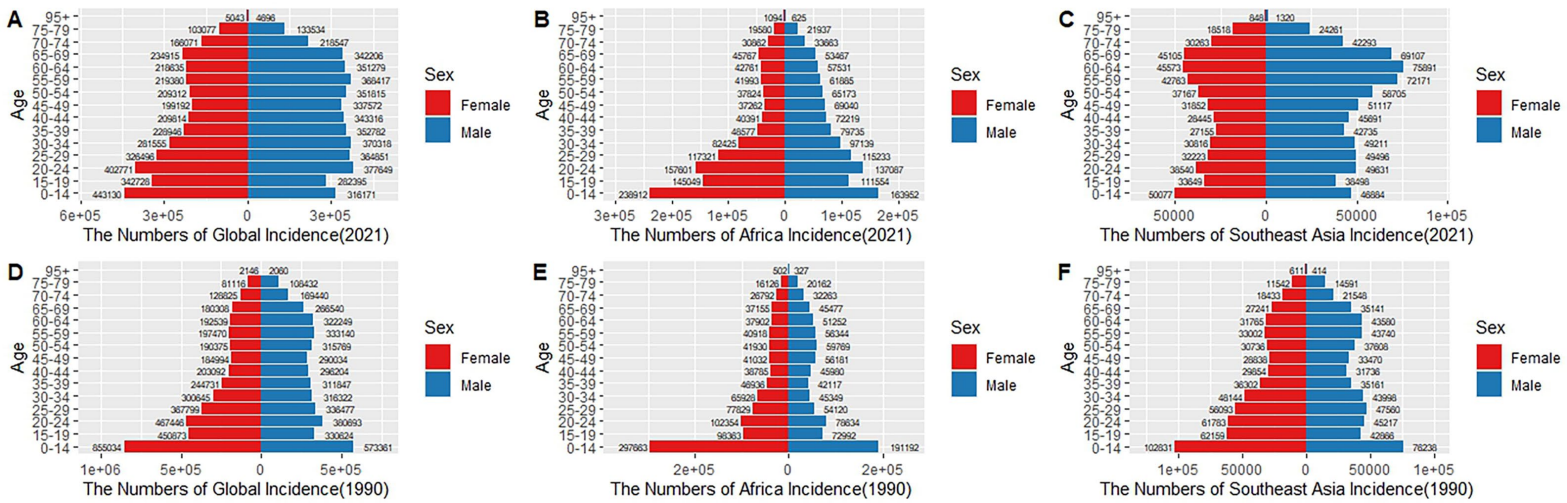
Sex- and age-specific distribution of global, African, and Southeast Asian TB incidence in 1990 and 2021. **(A–C)** 2021; **(D–F)** 2021.

The age-sex distribution of tuberculosis incidence in absolute numbers globally, in Africa, and in Southeast Asia for the years 1990 and 2021 is presented in Figure 7. Overall, males had consistently higher incidence numbers across almost all age groups and regions in both years. Globally and regionally, the largest incidence numbers were observed among young and middle-aged adults (15–54 years), with notable reductions in absolute case numbers from 1990 to 2021 across all groups. Specifically, global incidence significantly declined among younger populations, although the relative distribution remained similar. In Africa, incidence remained particularly high among younger age groups (15–34 years) but showed considerable absolute reductions over time. Southeast Asia experienced substantial declines in total numbers, particularly among younger adults (20–34 years). Across all three regions, the incidence among individuals aged 65 and above was relatively low but showed less pronounced declines compared to younger groups. The sex disparity persisted consistently, with males reporting notably higher incidence across all age categories and regions.

### Gender disparities in the burden of TB in full-age cases and age-standardized rates groups in 1990 and 2021

**Figure 8:**
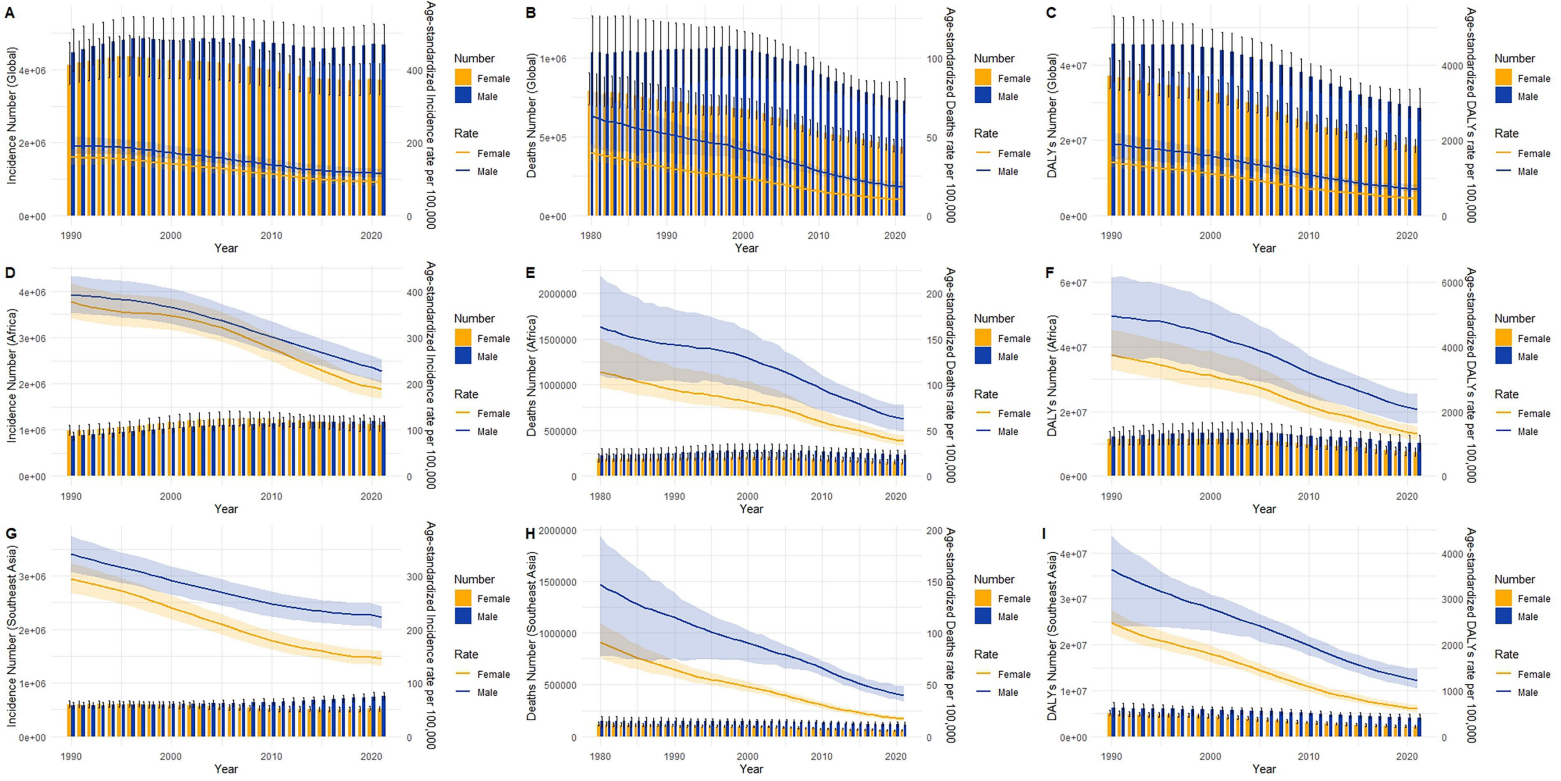
Trends in Global, Africa and Southeast Asia TB burden from 1990 to 2021, stratified by sex. **(A,D,G)** Incidence; **(B,E,H)** Deaths; **(C,F,I)** DALYs.

The trends in absolute numbers and age-standardized rates of tuberculosis incidence, deaths, and DALYs from 1990 to 2021 globally, in Africa, and in Southeast Asia, stratified by sex, are presented in Figure 8. Globally (Figures 8A–C), although absolute incidence numbers remained relatively stable over the studied period, age-standardized incidence rates displayed a slight decreasing trend. Deaths and DALYs showed more notable reductions, particularly after the early 2000s, with males consistently exhibiting higher rates and absolute numbers compared to females. In Africa (Figures 8D–F), absolute incidence numbers remained stable or slightly increased, while age-standardized incidence rates significantly declined over time. African mortality and DALYs demonstrated pronounced declines in both absolute numbers and age-standardized rates, again with males having notably higher burdens. Southeast Asia (Figures 8G–I) exhibited clear declines across all measures, particularly for deaths and DALYs, in both absolute numbers and rates, with consistent male predominance. The declining trends were most prominent for DALYs and deaths across all three regions, whereas incidence rates showed less dramatic reductions, highlighting improvements in treatment and management rather than a marked decrease in new tuberculosis infections.

### Projected trends in tuberculosis incidence and mortality across Africa, Southeast Asia, and globally

**Figure 9:**
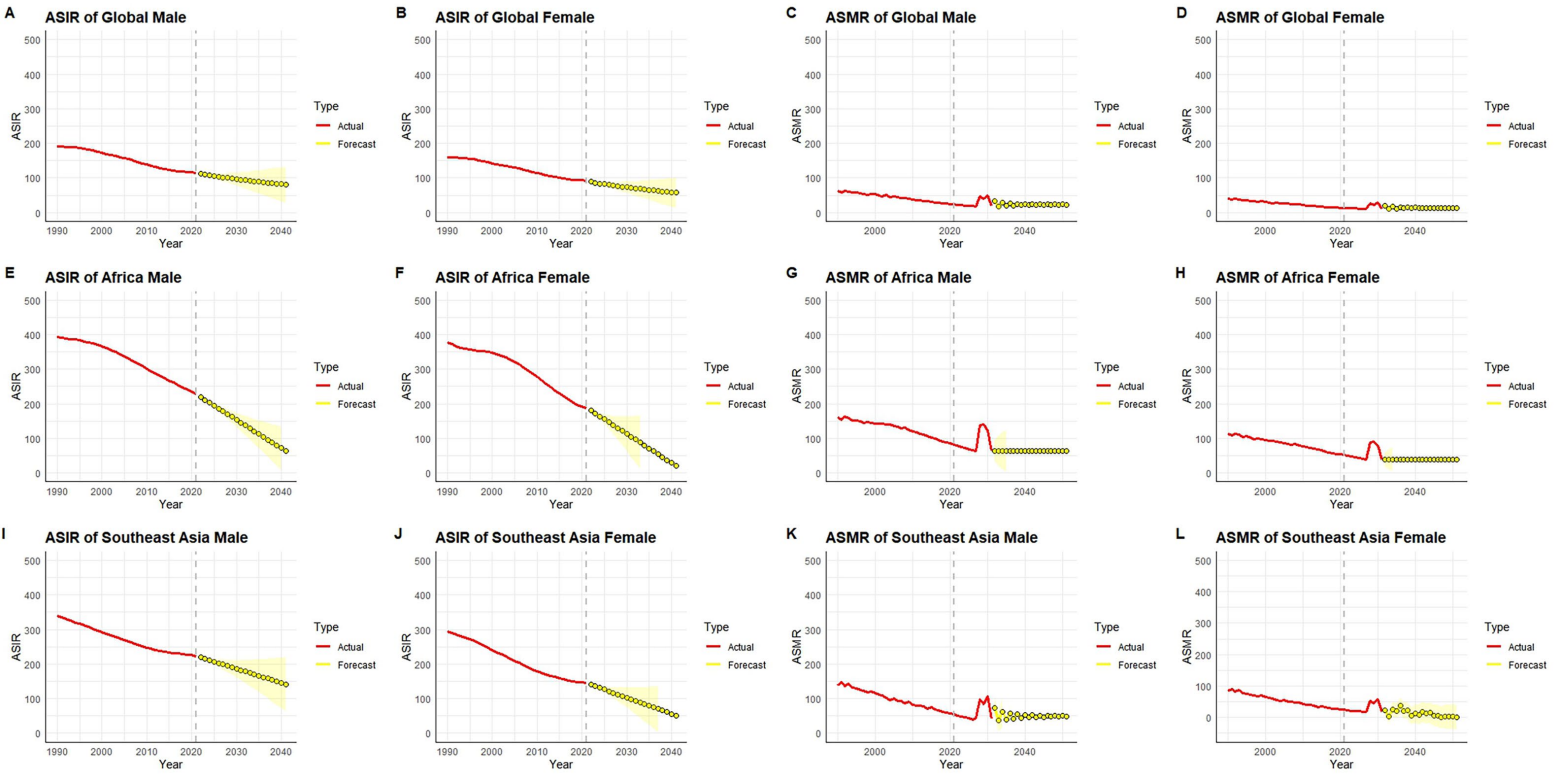
Observed and forecasted trends of age-standardized TB incidence rate (ASIR) and mortality rate (ASMR) from 1990 to 2040, by sex and region. **(A–D)** Global; **(E–H)** Africa; **(I–L)** Southeast Asia.

Between 1990 and 2021, the ASIR and ASMR demonstrated consistent downward trends globally, in Africa, and in Southeast Asia, with further reductions projected through 2040 (Figures 9A–L). Globally, both male and female ASIRs showed a gradual decline over the observed period (Figures 9A,B), with projections indicating continued reductions to below 100 per 100,000 by 2040. A similar trend was observed for global ASMRs (Figures 9C,D), which remained relatively low throughout the historical period and are forecast to further decrease, with minimal sex difference after 2030.

In Africa, ASIRs for both males and females declined more steeply than the global average (Figures 9E,F), with projected values expected to fall below 100 per 100,000 by 2040, indicating sustained progress. However, ASMRs in Africa (Figures 9G,H) displayed more variability, including a noticeable temporary fluctuation around 2030 in both sexes, followed by stabilization at low levels. Despite overall improvements, ASMRs in Africa remained slightly higher than those in global and Southeast Asian contexts.

In Southeast Asia, the ASIRs (Figures 9I,J) followed a pattern similar to Africa, with consistent declines and projections showing continued reductions through 2040. ASMRs (Figures 9K,L) decreased steadily from 1990 onward, with a sharp reduction in male mortality rates and relatively stable but lower levels in females. By 2040, both ASMR and ASIR in Southeast Asia are forecast to reach levels comparable to global averages.

Taken together, these projections reflect sustained progress in reducing incidence and mortality rates across all regions and sexes, with the steepest improvements observed in Africa and Southeast Asia. Although current disparities persist—particularly in mortality—the anticipated convergence in ASMRs and ASIRs across regions by 2040 suggests narrowing geographic and sex-based gaps in disease burden.

## Discussion

The TB remains a significant public health concern, particularly in low-resource settings (14). In this study, we conducted a comprehensive evaluation of TB incidence, mortality, and DALYs over the past 30 years in Africa, Southeast Asia, and globally, using the GBD database. We compared the differences in TB burden by age and gender, with a particular focus on regional trends and predictive analyses.

Our temporal analysis highlights diverging patterns across key indicators. From 1990 to 2021, the ASIR, ASMR, and ASDR exhibited significant trends across different regions. Overall, there was a marked decrease in ASIR, ASMR, and ASDR, reflecting improvements in TB control measures (14–16).

In Africa, ASIR and ASMR declined significantly, particularly after 2000. While ASDR also declined steadily, albeit less sharply than in South-East Asia. These reductions likely reflect enhanced healthcare resources, strengthened prevention and control measures, and expanded international cooperation aimed at mitigating the TB burden (17, 18). Despite these gains, the absolute number of TB cases in Africa rose by 22.63% between 1990 and 2021, underscoring persistent challenges in disease control. However, this increase is largely attributable to rapid population growth and demographic aging, which elevate absolute case counts even as age-standardized risks decline. The World Health Organization (WHO) and the International Union Against Tuberculosis and Lung Disease (IUATLD) have developed comprehensive strategies to reduce TB mortality, morbidity and transmission. It is therefore imperative that governments prioritize TB control, strengthen the management of co-infected patients, and implement precise, evidence-based control plans. This effort must be supported by adequate funding to maintain community capacity for generating demand for preventive therapies and delivering integrated services (19).

In contrast, South-East Asia achieved substantial declines in both rates and counts. Southeast Asia exhibited the most pronounced reductions in TB burden, with ASIR, ASMR and ASDR all declining markedly—particularly between 2000 and 2010—during which ASIR and ASDR fell by over 40%, underscoring the impact of intensified public-health interventions (20). Nevertheless, Southeast Asia continues to shoulder a substantial share of the global TB burden, with incident TB cases increasing by 8.39% between 1990 and 2021—a trend consistent with previous studies (20)—underscoring that TB remains a major public health challenge in the region.

The study also revealed distinct age- and gender-specific patterns in TB burden. Globally, incidence peaked in the 25–49 age group, with rates consistently higher among men than women— a trend mirrored in Africa and South-East Asia, though gender disparities were most pronounced in the African region. These findings align with prospective cohort data from Ethiopia, which similarly documented persistent TB incidence and prevalence in young populations (21). In Africa, the TB burden shifted toward older populations, with the highest incidence rates occurring in the 70–74 age group in 1990 and the 80–84 group in 2021. This trend reflects demographic aging and the growing vulnerability of older adults to TB. A prior national study similarly identified the older adult—and individuals with comorbidities or socioeconomic disadvantage—as high-risk groups (22). We recommend designing targeted intervention plans for high-risk populations—strengthening health monitoring, enhancing clinical management and expanding access to preventive services—while simultaneously promoting health education and optimizing resource allocation. A crucial component of our study is the ARIMA-based forecast of TB trends, which projects that ASIR and ASDR in Africa, South-East Asia and globally will continue their downward trajectories through 2040. These forecasts reinforce the imperative to sustain and intensify effective public-health measures and to expand healthcare access if further reductions in the TB burden are to be achieved (23). These demographic shifts inform our targeted intervention recommendations.

However, our ARIMA forecasts also identify a transient fluctuation in the global ASDR around 2030—a feature that is attenuated in Africa and South-East Asia but more pronounced at the global level. Importantly, this anomaly does not alter the overall downward trajectory, underscoring the sustained impact of current TB control efforts worldwide.

The decline in TB burden can be attributed to various factors, including improved living standards (24), widespread access to healthcare (25), and the extensive use of effective treatments. Nonetheless, the persistent high prevalence and incidence rates in certain regions underscore ongoing challenges such as poverty (26), limited healthcare resources (27), and genetic susceptibility to TB (28).

Our trend analyses allow a crude appraisal of the effectiveness of major tuberculosis control measures implemented since the early 1990s. The steep global decline in age-standardized mortality and DALY rates that began after 2000 coincides with the scale-up of DOTS/Stop-TB, wider access to rifampicin-based short-course chemotherapy and—especially in Africa—the rapid expansion of antiretroviral therapy, which reduced HIV-associated TB deaths. The plateauing of prevalence in South-East Asia after 2015, despite declining mortality, suggests that treatment success has improved survival without yet curbing ongoing transmission, underscoring the need for intensified active case-finding and infection-control measures. These inferences must, however, be interpreted in light of the inherent limitations of GBD data. First, notification and vital-registration systems remain incomplete in many high-burden countries, leading to underreporting of both incident cases and TB-attributable deaths; GBD compensates with covariate-based modeling, but residual downward bias is possible. Second, misclassification can occur when HIV-positive individuals are recorded as dying from AIDS rather than TB, or when multidrug-resistant TB is coded under non-TB respiratory conditions, potentially distorting regional mortality patterns. Third, revisions in case definitions and diagnostic practices over 30 years introduce temporal discontinuities that may either exaggerate or dampen true trends. Finally, the use of aggregated regional estimates masks within-country heterogeneity, limiting causal attribution of program effects. Despite these caveats, the concordance we observe between policy milestones and directional changes in key indicators strengthens confidence that the interventions implemented to date have contributed materially to the global decline in TB burden while highlighting gaps that future strategies must address.

Taken together, our three-decade trends indicate that the world remains off-track for the End TB Strategy’s interim and 2030 targets. Between 2015 (the baseline year used by WHO) and 2021, the global age-standardized TB incidence rate fell by only ≈6%, far short of the 2020 milestone of a 20% reduction, while mortality dropped by ≈12%, well below the 35% target. Translating our ARIMA forecasts to 2030 suggests that, at the current pace, annual declines in incidence would need to accelerate from ~1.7 to >4% per year, and mortality declines from ~3.1 to >7% per year, to achieve the Strategy’s goal of a 90% reduction in deaths and an 80% reduction in incidence.

Regionally, sub-Saharan Africa has made commendable progress in cutting mortality—thanks to DOTS expansion, antiretroviral therapy and improved case management—yet still faces rising absolute DALYs, underscoring the need to scale up preventive therapy and integrate TB–HIV services. South-East Asia shows a sharper fall in deaths but a plateau in prevalence after 2015, implying that enhanced active case-finding, rapid molecular diagnostics and treatment-adherence support are essential to interrupt ongoing transmission. These region-specific gaps highlight where intensified investment and tailored interventions are required if the End TB milestones are to be met globally.

This study has several limitations. First, all estimates are derived from the Global Burden of Disease (GBD) 2021 modeling framework, and their accuracy is constrained by the completeness and quality of the underlying data sources, which remain sparse in many low- and middle-SDI settings; under-ascertainment in these areas may therefore lead to an underestimation of the true TB burden. Second, the methodological divergence between GBD and the WHO Global Tuberculosis Report has important programmatic consequences. WHO estimates rely principally on country-reported notifications that are subsequently adjusted by Bayesian models and expert judgment; when surveillance is interrupted—as during the COVID-19 pandemic, which produced an 18% drop in global notifications in 2020—these figures can decline sharply. By contrast, the GBD framework triangulates notifications with mortality-registration data, prevalence surveys, and covariate modeling, yielding a lower but more stable 2021 estimate of 9.4 million incident cases and 1.35 million deaths, versus WHO’s 10.6 million and 1.4 million. Such divergence underscores surveillance blind spots and suggests that apparent gains based solely on notifications may be artifacts of under-reporting. Restoring diagnostic capacity, expanding Xpert/Ultra coverage, and conducting prevalence surveys in high-burden countries will be essential to reconcile these series and accurately track End-TB progress. Third, potential biases arising from disease misclassification and miscoding in vital-registration or surveillance systems could further affect accuracy. In addition, GBD 2021 models only HIV-negative tuberculosis and excludes HIV-associated TB; therefore, in settings with high HIV prevalence, this study may underestimate the overall burden of TB deaths and DALYs. Finally, because the analysis is performed at global and broad regional levels, the results may not be directly transferable to individual countries, where socio-economic, environmental and health-system contexts differ.

By thoroughly analyzing TB trends, regional differences, and future projections, we hope this study provides valuable insights for global health policy formulation, promoting more effective TB control measures and further mitigating TB’s threat to global health. Continued efforts are necessary to address the persistent challenges in controlling TB, particularly in low and middle SDI areas, by improving access to healthcare services and resources to achieve further reductions in TB-related morbidity and mortality.

## Conclusion

This comprehensive analysis of the global TB burden from 1990 to 2021 demonstrates substantial reductions in age-standardized incidence, mortality and DALY rates—most notably in South-East Asia—while revealing that absolute case counts continue to rise in Africa despite improvements in standardized metrics. Our findings expose enduring regional disparities and underscore the necessity of targeted, context-specific interventions. Predictive analyses project further declines in TB rates worldwide through 2040, yet persistent obstacles—poverty, constrained healthcare infrastructure and genetic susceptibility—threaten to slow progress. Together, these results highlight the imperative of sustained, coordinated global efforts and tailored strategies to accelerate declines in TB morbidity and mortality, especially in high-burden regions.

## Data Availability

The datasets presented in this study can be found in online repositories. The names of the repository/repositories and accession number(s) can be found at: http://ghdx.healthdata.org/gbd-results-tool.

## Footnotes

^1^ https://vizhub.healthdata.org/gbd-results/
