# Supplementary material for: Decadal trends and regional disparities in tuberculosis burden: a comprehensive analysis of global, African, and Southeast Asian data from the GBD 1990–2021
Source: Front Public Health. 2025 Aug 4;13:1467509. doi: 10.3389/fpubh.2025.1467509 (PMC12358351; doi:10.3389/fpubh.2025.1467509)
Supplement: Supplementary file 1 [file Table_1.docx]

# **Supplementary Table S1. Diagnostic indicators for ARIMA models fitted to incidence and death trends of tuberculosis across regions and sexes.**

| **Region** | **Sex** | **Type** | **Model** | **AIC** | **AICc** | **BIC** | **Sigma²** | **LogLik** | **ME** | **RMSE** | **MAE** | **MPE** | **MAPE** | **MASE** | **ACF1** |
| --- | --- | --- | --- | --- | --- | --- | --- | --- | --- | --- | --- | --- | --- | --- | --- |
| **Global** | Male | Incidence | ARIMA(3,1,0) | 33.57 | 35.11 | 39.31 | 0.1285 | -12.79 | -0.082 | 0.335 | 0.268 | -0.047 | 0.179 | 0.111 | 0.06 |
| **Global** | Female | Incidence | ARIMA(3,1,0) | 43.21 | 44.75 | 48.95 | 0.1771 | -17.61 | -0.095 | 0.394 | 0.278 | -0.066 | 0.238 | 0.127 | 0.056 |
| **Global** | Male | Death | ARIMA(2,1,0) | 271.83 | 272.48 | 276.98 | 39.63 | -132.92 | -1.004 | 6.067 | 2.912 | -4.732 | 8.841 | 0.851 | 0.111 |
| **Global** | Female | Death | ARIMA(2,1,0) | 230.18 | 230.83 | 235.33 | 14.36 | -112.09 | -0.674 | 3.652 | 1.868 | -5.179 | 9.583 | 0.849 | 0.094 |
| **Africa** | Male | Incidence | ARIMA(0,2,1) | 43.4 | 43.85 | 46.2 | 0.2351 | -19.7 | -0.152 | 0.462 | 0.317 | -0.047 | 0.104 | 0.059 | -0.08 |
| **Africa** | Female | Incidence | ARIMA(0,2,2) | 65.37 | 66.3 | 69.58 | 0.4423 | -29.69 | -0.047 | 0.622 | 0.471 | -0.015 | 0.182 | 0.077 | 0.004 |
| **Africa** | Male | Death | ARIMA(0,1,0) | 343.5 | 343.6 | 345.21 | 242.6 | -170.75 | -2.287 | 15.388 | 6.639 | -3.472 | 6.578 | 0.977 | 0.106 |
| **Africa** | Female | Death | ARIMA(0,1,0) | 309.66 | 309.76 | 311.37 | 106.3 | -153.83 | -1.726 | 10.185 | 4.754 | -3.908 | 7.262 | 0.977 | 0.109 |
| **Southeast Asia** | Male | Incidence | ARIMA(0,2,0) | 69.03 | 69.17 | 70.43 | 0.5549 | -33.51 | 0.03 | 0.721 | 0.382 | 0.008 | 0.152 | 0.101 | -0.026 |
| **Southeast Asia** | Female | Incidence | ARIMA(0,2,1) | 48.7 | 49.15 | 51.51 | 0.2677 | -22.35 | -0.014 | 0.493 | 0.279 | -0.003 | 0.156 | 0.058 | 0.035 |
| **Southeast Asia** | Male | Death | ARIMA(2,1,0) | 333.35 | 334.0 | 338.49 | 177.2 | -163.67 | -2.137 | 12.827 | 6.154 | -4.489 | 8.696 | 0.793 | 0.123 |
| **Southeast Asia** | Female | Death | ARIMA(4,1,0) with drift | 288.68 | 291.15 | 298.96 | 53.73 | -138.34 | 0.063 | 6.786 | 3.437 | -0.705 | 7.958 | 0.66 | 0.009 |

Note: AIC = Akaike Information Criterion; AICc = corrected AIC; BIC = Bayesian Information Criterion; ME = Mean Error; RMSE = Root Mean Square Error; MAE = Mean Absolute Error; MPE = Mean Percentage Error; MAPE = Mean Absolute Percentage Error; MASE = Mean Absolute Scaled Error; ACF1 = First-order autocorrelation of residuals. Sigma² = estimated variance of residuals; LogLik = log likelihood value of the model.
